# Opioid Room of Horrors: a simulation approach to strengthen drug administration safety

**DOI:** 10.1136/bmjoq-2025-003728

**Published:** 2025-12-25

**Authors:** Sophia Hannou, Cristina Nicorici, Wanda Bosshard, Pierre Voirol, Farshid Sadeghipour, Nancy Perrottet, Chantal Csajka

**Affiliations:** 1 Center for Research and Innovation in Clinical Pharmaceutical Sciences, Lausanne University Hospital and University of Lausanne, Lausanne, Switzerland; 2 Department of Pharmacy, Lausanne University Hospital, Lausanne, Switzerland; 3 Service of Geriatric Medicine and Geriatric Rehabilitation, Department of Medicine, Lausanne University Hospital, Lausanne, Switzerland; 4 School of Pharmaceutical Sciences, University of Geneva, Geneva, Switzerland; 5 Institute of Pharmaceutical Sciences of Western Switzerland, University of Geneva, University of Lausanne, Geneve, Switzerland

**Keywords:** Human error, Medication safety, Patient safety, Quality improvement, Continuing education, continuing professional development

## Abstract

**Background and objectives:**

Medication administration errors (MAEs) are frequent and preventable. While the five rights (5R) rule and the double-check are standard practices for safe drug administration, incidents still occur. MAEs involving high-risk drugs such as opioids are a major concern, especially in older patients. To address this, a practical, error-driven training was developed through an opioid Room of Horrors (RoH) to reinforce the 5R rule and the double-check procedure and map risks within the opioid medication-use process, thereby improving the safety of opioid administration. The secondary objective was to evaluate participant satisfaction with the training.

**Method:**

The opioid RoH integrates four errors and four hazards hidden in the opioid medication-use process. Participants, working in pairs, were asked to prepare and administer an opioid to a fictitious patient. Two assessors recorded the number of errors detected and hazards avoided. During the debriefing, assessors reviewed and explained these items to the pair of trainees combined with a refresh on the 5R rule and the double-check process. Detection and avoidance rates were analysed using descriptive statistics. Participants assessed the training through a satisfaction questionnaire.

**Results:**

A total of 86 sessions were conducted, involving 172 participants including nurses, nurse assistants and physicians from a geriatric department. Participants detected errors such as wrong patient (60%), expired drugs (63%), incorrect strength or galenic form (55%) and documented allergy (55%), while most hazards were avoided, except for the correct device, which was used in only 65% of cases. Double-check performance was inefficient mainly focusing on the dose check. Satisfaction was high (9.2/10), and 73% of participants reported a knowledge gain.

**Conclusions:**

The opioid RoH is an effective training to refresh and emphasise the rigorous application of the 5R rules and the double-check procedure to reduce MAEs. Additionally, this simulation showed persistent gaps such as patient identification and double-check failures, highlighting the need to strengthen safety practices through continuous training and institutional-level system improvements in medication administration safety.

**WHAT IS ALREADY KNOWN ON THIS TOPIC**

- The five rights rule and the double-check are tools widely known by healthcare professionals to secure medication administration, yet errors can sometimes occur.

**WHAT THIS STUDY ADDS**

- This opioid Room of Horrors is an effective training to increase clinicians’ awareness in applying safe medication administration practices and to identify gaps in opioid preparation and administration.

**HOW THIS STUDY MIGHT AFFECT RESEARCH, PRACTICE OR POLICY**

- This training enables hospitals to reveal system vulnerabilities linked to opioid preparation and administration. Its format is transferable to other institutions and can be adapted to other high-risk medications, offering opportunities for broader evaluation. Embedding this approach into institutional education programmes may help strengthen medication safety culture and reduce preventable medication-related harm.

## Introduction

 A medication error is an unintended and preventable event leading to patient harm or has the potential to do so. It can occur at any stage of the medication process, including prescription, preparation, administration and monitoring.^1 2^ Nearly half of medication errors occur during the administration stage, with error rates ranging from 15% to 26% of administered doses.^3^,^5^ The administration stage is the last barrier in the medication circuit, and therefore requires an increased vigilance to ensure safe drug administration, especially for high-risk medication such as opioids due to their high potential for harm.^6^ Opioid administration error represents 8% of adverse drug events in hospital.^7^ This incidence is expected to rise due to the increase in opioid prescriptions in older adults.^8^ The highest prevalence of preventable medication harm is observed in geriatric units, a population at higher risk of comorbidity, polypharmacy and frailty.^9 10^ These preventable errors underscore the need for action to mitigate opioid-related harm through preventive measures.^11^,^13^

Different strategies have been deployed worldwide to reduce the risk of preventable medication administration errors (MAEs).^14 15^ One of them is a systematic application of the five rights (5R) rule which ensures that the right drug is administered at the right dose, via the right route, at the right time, to the right patient, as a standard for safe administration practice. Another complementary approach is to apply an independent double-check process before administration by two healthcare professionals who independently perform the same check,^16^ which is more effective in detecting errors than a single check.^17^ Yet, due to a lack of human resources or time constraints, the double-check is not systematically applied before each administration, except for high-risk drugs such as chemotherapy, some injectable medicines or opioids.^6 18 19^

In the geriatric department of our hospital, the 5R rule must be applied before each drug administration, and an additional independent double-check is mandatory specifically for opioids. Yet, in 2022, MAE involving opioids accounted for 21% of all reported MAE, with an increasing number of incidents reported to the voluntary, anonymous, electronic safety reporting database. This raises concerns about the effective and optimal use of these safety checks. A new teaching approach was introduced to enhance awareness among healthcare professionals and promote the effective application of these safety tools.

Simulation training programmes are considered effective approaches for acquiring skills to prevent medication errors across the medication-use process.^20 21^ One effective type of simulation designed to enhance situational awareness is the ‘Room of orrors’ (RoH).^22^,^24^ In this exercise, educators create a simulated environment that intentionally incorporates multiple patient safety errors or hazards. Participants are tasked with identifying these errors, fostering a heightened awareness of potential risks in their practice. The ultimate goal is to equip participants with the skills and insights needed to proactively address safety threats and implement measures to improve patient safety.^24^ Thus, an opioid RoH was developed as a focused initiative to raise awareness and improve the application of safety practices with the purpose of reducing opioid MAE and ultimately improving patient safety and quality of care.

This project aimed to design an innovative, practical and error-driven RoH for opioids. The objectives were to reinforce drug administration safety by strengthening adherence to the 5R rule and the double-check performance, and to map risks within the opioid circuit. Another objective was to evaluate participant satisfaction with this pedagogical approach.

## Methodology

### Elaboration of the opioid RoH

A clinical pharmacist and a nurse led the project, developing a scenario involving a fictitious patient that reflects cases commonly encountered in the geriatric unit. They created four errors and four hazards based on incidents of opioid administration reported in the geriatric department at Lausanne University Hospital between August 2021 and August 2022. These incidents were selected for their recurrence and thorough documentation. The scenario, along with the errors and hazards, was then approved by an expert group from the geriatric department, which included one quality expert, one geriatrician, two nurses and one clinical pharmacist. The simulation room was set up to closely resemble a real clinical environment. It included a mannequin with a fictitious patient wristband and subcutaneous access, a computer displaying a mock electronic medical record (EMR), a medicine cabinet containing fictitious opioid drugs, a nurse trolley with medical devices including syringes, and an opioid file with a stock-tracking sheet. A dedicated room within the geriatric unit was designated for the training.

#### Scenario

Participants were asked to prepare and administer an opioid to the fictitious patient based on the following scenario and prescription according to the scenario and the prescription detailed in box 1.

Box 1 **Scenario description**

“Mr. X was admitted to the geriatric rehabilitation center two days ago for rehabilitation following a left hip replacement surgery that occurred ten days earlier. He has multiple comorbidities and allergies. This morning, after returning from his physiotherapy session, he again reported pain in his left hip, rating it 8 out of 10, and requested painkillers for relief. It is now 9:00 a.m.”

Prescription in the medical record:

‘Morphine hydrochloride oral solution 10 mg/mL: 3 mg=0.3 mL every 4 hours (8:00, 12:00, 16:00, 20:00, 12:00, 4:00).

Morphine hydrochloride oral solution 10 mg/mL: 3 mg=0.3 mL up to 6 times/day if needed’.

#### Errors and hazards in the opioid RoH

Four errors and four hazards were introduced in the room, covering different steps of the opioid medication-use process. Both errors and hazards were designed to be either detected or avoided if the 5R rule and the double-check process were applied effectively. These are presented in table 1.

**Table 1:** List of errors and hazards introduced in the opioid Room of Horrors

Errors and hazards	Item	Explanation
Errors		
Error 1	Wrong patient identification	The name, first name and date of birth on the patient’s wristband differ from those in the EMR: subtle differences in the name, first name, and date of birth
Error 2	Wrong drug—strength or galenic form	The prescribed morphine, 10 mg/mL oral solution, is missing from the opioid cabinet; only a 1 mg/mL oral solution and a 10 mg/mL injectable solution are available.
Error 3	Wrong drug—expired drug	An expired drug is administered to the patient; the opened bottle available in the cabinet has exceeded its shelf-life.
Error 4	Wrong drug—documented allergy	Morphine is administered despite a documented opioid allergy in the patient’s EMR.
Hazards		
Hazard 1	Wrong device	A transparent syringe, which is a colour code for parenteral use, is selected instead of a violet syringe, used for oral or enteral route.
Hazard 2	Wrong volume	The volume withdrawn does not match the prescribed dose.
Hazard 3	Wrong route of administration	The medication is administered via the subcutaneous route instead of the prescribed oral route.
Hazard 4	Wrong time	The timing of the last opioid dose administered is not verified, leading to a risk of inappropriate dosing intervals.

#### Double-check performance evaluation

Once the preparation was completed, double-check performance was evaluated across seven items: verifying the five elements of the 5R rule independently, using the original prescription and the drug used for preparation, and speaking clearly and aloud.

### Set-up of the simulation

The structure of the training session adhered to recommended best practices for simulations, which include a briefing phase, the exercise itself and a debriefing phase.^25^ Each 30 min training session involved a pair of healthcare professionals (nurse/nurse, nurse/healthcare assistant or physician/physician) working in the geriatric department and was assessed by the clinical pharmacist and the nurse who developed the training. During the briefing, participants were introduced to the objectives, the instructions and the clinical scenario used for the exercise. In the exercise phase, participants prepared and administered an opioid dose while educators evaluated error detection, hazard avoidance and double-check performance using a standardised assessment form. The debriefing provided structured, individualised feedback and reinforced adherence to safe medication administration practices. Online supplemental appendix 1 provides a detailed description of the simulation process.

### Satisfaction questionnaire

At the end of the session, participants were invited to complete an anonymous evaluation survey. It included a satisfaction question (Likert scale from 0 to 10), a self-assessment of their knowledge of the 5R rule and the double-check process before and after the training (scale: none, low, medium, high), and an open-ended section for comments. Demographic data, including age, gender, educational background and current medical ward, were also collected.

### Data analysis

The number and percentage of errors and hazards were calculated for each pair of participants, stratified by type, and summarised using descriptive statistics (proportions, median and IQR). The same analysis was applied to the seven items related to the double-check performance. Differences in error, hazard and double-check items rates between nurses, healthcare assistants and physicians were assessed using a two-sample test of proportions. A significance level of p<0.05 was applied. Satisfaction levels and changes in self-reported knowledge before and after the training were described using summary statistics. All analyses were conducted using Stata (Statistical Software, StataCorp, release 18).

### Patient and public involvement

Patients and members of the public were not involved in the design, conduct, reporting or dissemination of this research.

## Results

The elaboration of the opioid RoH scenario took 1 month and required approximately 15 hours of work. A total of 172 healthcare professionals from the geriatric department at Lausanne University Hospital participated in the training, which provided a practical refresher of the 5R rule and the double-check process. The training was delivered through 86 sessions held over 7 days between September 2022 and June 2024, five sessions in the geriatric rehabilitation unit and two in the geriatric acute care unit, covering 70% of the permanent staff. A total of 169 participants completed the post-training survey. Participants’ characteristics are presented in table 2.

**Table 2:** Participant characteristics (n=172)

Variable	n (%)
Educational background	
Nurse	86 (50%)
Healthcare assistant	38 (22%)
Physician	48 (28%)
Medical ward	
Geriatric rehabilitation centre	136 (79%)
Geriatric acute unit	36 (21%)
Age*	
20–29	68 (40%)
30–39	56 (33%)
40–49	25 (15%)
50–59	16 (9%)
60–65	2 (1%)
Not specified	2 (1%)
Gender*	
Women	130 (77%)
Man	36 (21%)
Not specified	3 (2%)

* Data based on the satisfactory questionnaire (n=169).

### Detection of errors, avoidance of hazards and double-check performance

The results for error detection, hazard avoidance and double-check performance are presented in table 3. Participants correctly identified or avoided a median of 6 out of 8 items. Detection rates varied depending on the type of error, with documented allergies and incorrect drug (strength or galenic form), each identified by only 55% of participants, expired drugs by 63%, and wristband/EMR name mismatches by 60%. In terms of hazard avoidance, 95% of participants withdrew the correct volume, and nearly all verified the timing of the last administration. However, only 65% used the appropriate violet oral syringe, and while 93% administered the drug orally, the remaining 7% delivered it via the incorrect subcutaneous route. The double-check performance was low, with a median of 1 out of 7 items performed. The dosage check was the most frequently completed item (48%), and all remaining items were completed by fewer than 30% of participants. Performance varied by professional role: nurses and healthcare assistants were more diligent in verifying expiry dates and selecting the correct syringe, while physicians were more likely to identify documented allergies. The double-check performance did not differ significantly between professional profiles.

**Table 3:** Percentages of errors detected, hazards avoided and double-check items performed

	All, n (%)	Nurses and healthcare assistants, n (%)	Physicians, n (%)	P value	95% CI
Participants	172	124	48		
Pairs of participants	86	62	24		
5 rights rule					
Errors detected (n;%)					
Patient—Wrong patient	52 (60%)	40 (65%)	12 (50%)	0.20	(–0.08 to 0.38)
Drug—Incorrect strength or galenic form	47 (55%)	35 (56%)	12 (50%)	0.61	(–0.17 to 0.30)
Drug—Expired drug	54 (63%)	43 (69%)	11 (46%)	0.048	(–0.00 to 0.46)
Drug—Documented allergy	47 (55%)	29 (47%)	18 (75%)	0.019	(–0.49 to 0.07)
Median errors detected (n=4)	2 (58%; IQR: 1.3–3)	2.5 (59%; IQR: 1.3–3)	2 (55%; IQR: 0–3)		
Hazard avoided (n;%)					
Dose—Use of an injectable syringe	56 (65%)	48 (77%)	8 (33%)	0.0001	(0.22 to 0.66)
Dose—Incorrect volume withdrawn	82 (95%)	60 (97%)	22 (92%)	0.31	(–0.07 to 0.17)
Route—Injectable administration	80 (93%)	58 (94%)	22 (92%)	0.74	(–0.10 to 0.14)
Time—Last administration not checked	85 (99%)	61 (98%)	24 (100%)	0.49	(–0.05 to 0.01)
Median hazards avoided (n=4)	4 (88%; IQR: 3–4)	4 (92%; IQR: 3–4)	3 (79%; IQR: 0–4)		
Median errors detected/hazard avoided (n=8)	6 (73%; IQR: 5–7)	6 (75%; IQR: 5–7)	6 (67%; IQR: 0–7)		
Double-check (n=7)					
Speak out loud	26 (30%)	20 (32%)	6 (25%)	0.52	(–0.14 to 0.28)
Look at the original prescription/drug	25 (29%)	20 (32%)	5 (21%)	0.31	(–0.09 to 0.31)
Check—Patient identity	21 (24%)	17 (27%)	4 (17%)	0.33	(–0.09 to 0.29)
Check—Drug	26 (30%)	21 (34%)	5 (21%)	0.23	(–0.07 to 0.33)
Check—Dosage	41 (48%)	33 (53%)	8 (33%)	0.09	(–0.03 to 0.43)
Check—Route of administration	8 (9%)	6 (10%)	2 (8%)	0.77	(–0.11 to 0.15)
Check—Time	10 (12%)	8 (13%)	2 (8%)	0.51	(–0.09 to 0.19)
Median double-check performance (n=7)	1 (26%; IQR: 0–3.8)	1 (29%; IQR: 0–4)	0 (19%; IQR: 0–4.3)		

### Participant training evaluation

**Figure 1:**
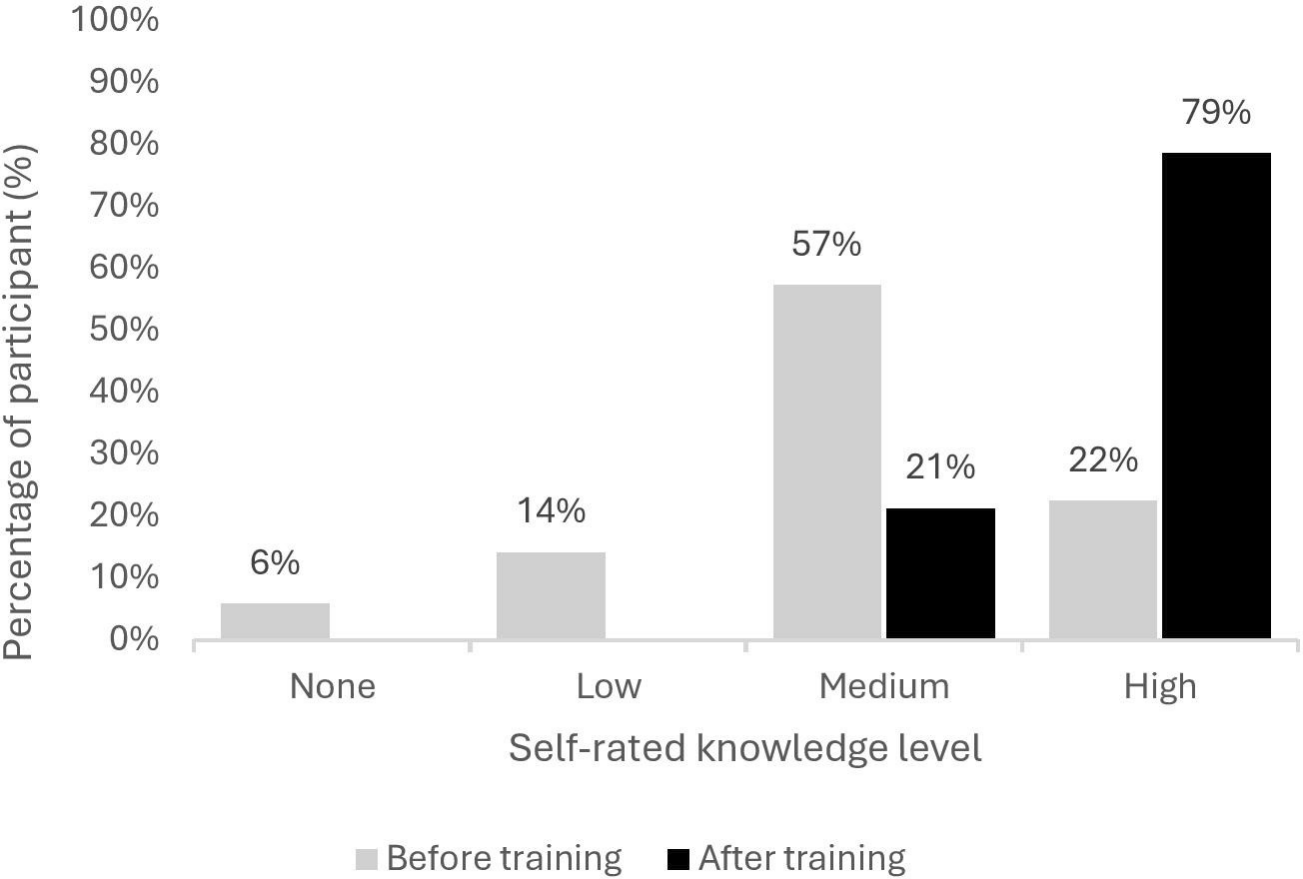
Self-rated knowledge of participants regarding the 5R rule and the double-check process before and after the training. 5R, five rights.

A total of 98% of participants (169/172) completed the anonymous, voluntary survey. Satisfaction with the training was high, with an average rating of 9.2 out of 10 across all professionals and 9.2, 9.3 and 9.1 for nurses, healthcare assistants and physicians, respectively. Before the training, only 22% of participants rated their knowledge level as high, increasing to 79% afterward (figure 1).

**Figure 2:**
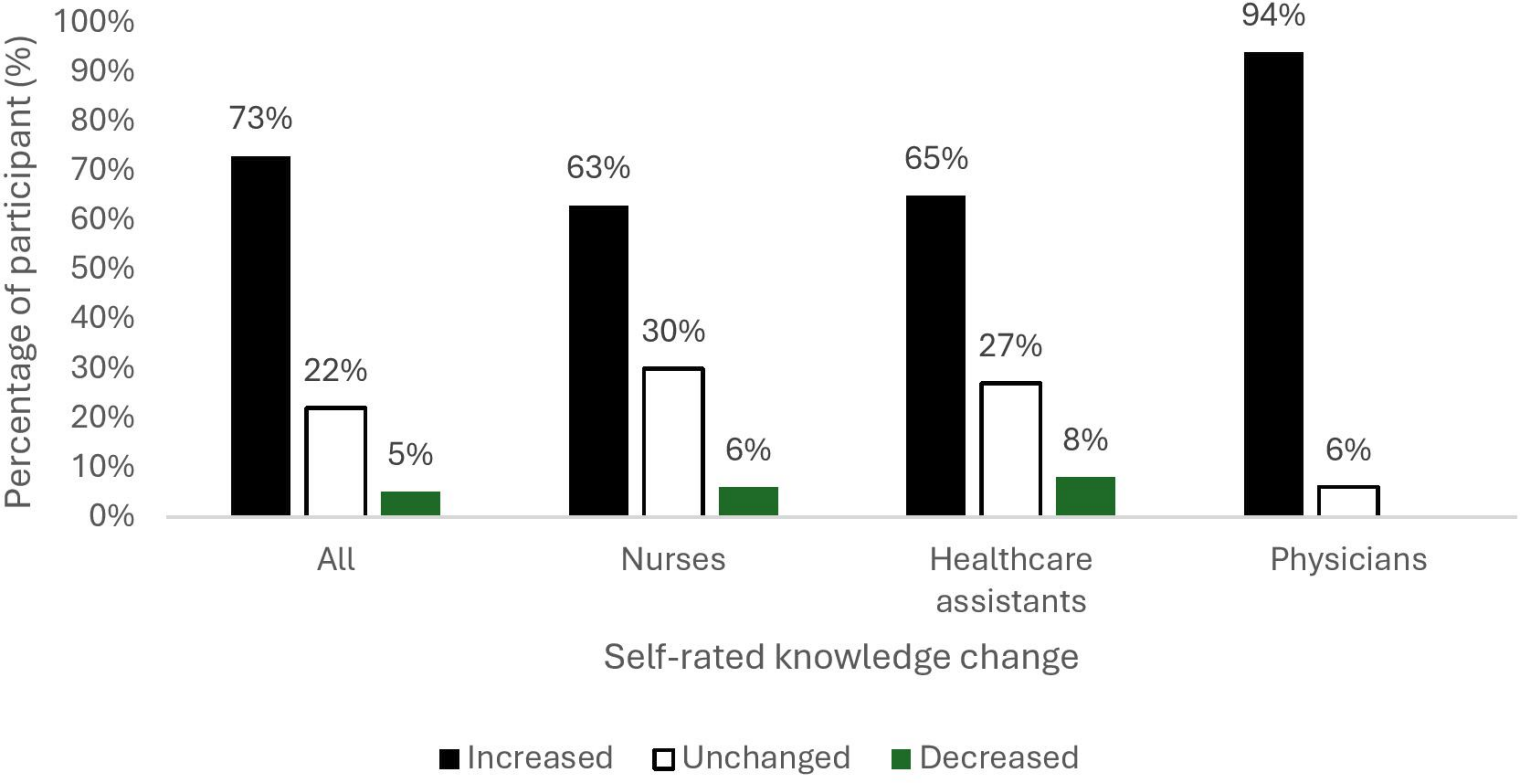
Self-rated change in knowledge after training by professional group.

Regarding knowledge gain, 73% of participants reported an upward progression in their self-assessed knowledge after the training (figure 2). The most frequent improvement was a shift from medium to high knowledge, reported by 53% of respondents. Other notable progressions included a shift from none or low to high (7%). About a quarter of participants reported no change in perceived knowledge level (including 18% at a high level), while only 5% reported a decrease from high to medium. Additionally, 41% of respondents provided comments, describing it as practical, focused on real errors, concise, relevant to their clinical practice, conveniently located within their unit, enjoyable, non-judgmental and effective in raising awareness about safety checks.

## Discussion

To the best of our knowledge, this training is the first RoH training to focus specifically on the preparation and administration of opioid drugs in a real-life setting, aimed at reinforcing the 5R rule and the double-check procedure. A large number of healthcare professionals from the geriatric department participated in the workshop. Numerous errors remained undetected, and the performance of the double-check was suboptimal. Participants expressed high satisfaction with the training, and about three quarters reported an improvement in their knowledge of these key safety practices. The opioid-focused RoH training appears to be an effective approach to enhance awareness of safety practices and to highlight the risks associated with opioid administration.

The opioid RoH was set up quickly, relying solely on internal resources, with a design focused on maximising learning efficiency. While scenario sources for such training may originate from standardised ready-to-use materials, literature reviews or expert input,^23 26^ the opioid RoH was uniquely built on real-life case scenarios drawn from MAE reports within the hospital’s geriatric department. This approach ensured that the scenarios were not only authentic but also highly relevant to realities faced by participants. In addition, incorporating the hospital’s EMR, standard patient wristbands, commonly used medications and typical prescribing patterns contributed to a high-fidelity simulation environment, known to enhance learning outcomes.^27 28^ In terms of team size, RoHs studies included two to eleven participants,^23^ but evidence suggests that limiting group size to fewer than six improved the effectiveness of simulation-based training.^29^ This training intentionally used pairs of participants to reflect real-life practices in opioid preparation and double-checking. Working in pairs enables educators to closely observe individual behaviours, explore participants’ thought processes, identify reasoning patterns that contribute to errors and adapt debriefing, which are key elements to deliver effective training.^21^ While RoHs training often includes an extensive number of hazards, sometimes exceeding 30, and spans multiple drug classes within the same scenario, this opioid-specific RoH focused only on morphine. By narrowing the scope, the training allowed a deeper exploration of opioid-related safety issues. This dynamic simulation, in which participants were assigned a concrete task, preparing and administering an opioid, helped maintain engagement by offering a focused, structured and clinically relevant exercise. Unlike broader RoHs, this targeted approach ensured that learning objectives remained clear, actionable and directly applicable to daily practice.^23^ Finally, the opioid RoH was intentionally designed to support interprofessional education, aimed at fostering shared understanding and awareness across healthcare roles, an essential step towards strengthening teamwork and ultimately improving patient safety.^30^ It provides physicians with valuable insight into the practical consequences of their prescriptions and helps them understand nurses’ challenges during medication preparation and administration. They may also be directly involved in the double-checking process. By encouraging participants to contribute beyond their defined responsibilities and emphasising individual and collective safety barriers, this training promotes an enhanced Swiss Cheese Model, which conceptualises adverse events as the alignment of failures across multiple system defenses, thereby fostering a more integrated safety culture.^31^ Simulation-based training has been shown to strengthen healthcare professionals’ awareness, confidence and critical thinking in safe medication practices. It also improves skills and clinical judgement, shapes attitudes and intentions regarding medication error reporting, and enhances knowledge related to advanced medication preparation. Together, these benefits highlight the value of experiential approaches such as the opioid Room of Horrors to support medication safety.^32^,^34^

The error detection and hazard avoidance rate of 73% aligns with the 34.5% to 90.1% detection rates observed in other RoH studies.^23^ This wide range may be attributed to differences in healthcare professionals’ profiles, group sizes and the types of errors assessed, which limits direct comparisons. In our study, only about half of participants selected the correct drug, while the others administered a different morphine (concentration or pharmaceutical form) available in the medication cabinet, constituting an MAE.^30^ This is concerning, as it may lead to dosing errors or wrong-route administration, both of which carry potential harm for the patient.

One of the most critical safety lapses observed was in patient identification, with a substantial number of participants failing to detect the mismatch between the patient’s wristband and the EMR; only 60% noticed this discrepancy. This rate was lower than the 80% identification performance reported in the literature.^20 35^ Such errors are particularly concerning, as administering a medication to the wrong patient can cause direct harm, while the intended patient may miss a necessary treatment. Similarly, the opioid allergy documented in the EMR was overlooked by nearly half of the groups. This finding is in line with previous studies on allergy detection, which report comparable rates.^24 36^ Alarmingly, some participants knowingly proceeded with administration, citing prior tolerance or doubting the accuracy of the allergy record, highlighting a serious gap in safety culture and communication between healthcare professionals. Lastly, expired medications were identified in 63% of the cases, consistent with previously reported rates of 57%–86%.^24 36 37^ Although expiration checks are a basic safety step, failures in interpreting expiry dates remain common and may expose patients to reduced efficacy or harmful degradation products. Overall, hazard avoidance was strong, with rates exceeding 90% for most items. However, syringe selection was an exception, with only 65% of participants using the correct oral syringe (violet). Despite the use of colour-coding to prevent wrong-route errors, this study highlights that the risk persists: six groups (7%) administered the drug subcutaneously instead of orally, likely influenced by the use of transparent syringes designed for parenteral routes.

The suboptimal performance of the double-check process reflected a lack of structured and systematic approach, despite institutional guidelines recommending that the controller independently repeat the entire preparation process from the beginning. A more consistent and rigorous application of this procedure might have prevented several undetected errors. This aligns with literature showing that double-checking often fails to prevent errors, primarily due to poor adherence rather than flaws in the concept itself.^16^

The anonymous survey showed an excellent average level of satisfaction, consistent with previous reports with simulation-based training in healthcare.^23^ A majority of participants perceived a knowledge increase after the session, highlighting the value of revisiting safety procedures through a new approach. Interestingly, 5% of participants reported decreased knowledge after the training, possibly due to overestimating their baseline knowledge or misjudging their pretraining and post-training assessments.

Based on these findings and participant feedback, several corrective measures and practical recommendations emerged. To address drug selection issues, participants were encouraged to proactively consult physicians when facing unavailability, formulation discrepancies or unclear prescriptions. They were also reminded not to proceed with administration in the presence of unresolved inconsistencies, such as documented allergy. While safety checks like patient identification and drug expiry verification are often performed instinctively, this training underscored the need to approach them with deliberate rigour. Participants were reminded that the use of incorrect devices may lead to serious route-related errors. The value of structuring the double-check to ensure it is systematic and reproducible was emphasised. Specifically, participants were encouraged to adhere strictly to the 5R rule at each step, to use the original prescription, the original drug used for the preparation and the final preparation during the verification, while speaking aloud each step to facilitate shared understanding. Participants were alerted not to focus solely on volume verification, which is often misused as a proxy for dose confirmation. Instead, volume checks should follow confirmation of the correct drug and concentration and be embedded in a comprehensive verification process.

This study has certain limitations. First, the independent double-check may be biased, as both participants performed the entire exercise simultaneously, limiting the possibility of a truly independent verification. Nevertheless, this set-up mirrors real-life practice, where the concept of independence in the double-checking is often imperfectly applied and remains an area for improvement.^38^ Future iterations of the simulation could refine the independent double-check process by having the first participant prepare the opioid medication alone, followed by the entry of a second participant to perform the verification. This adaptation would more accurately reproduce real-life clinical workflows and allow for a more realistic evaluation of the double-check procedure. Second, the evaluation of knowledge acquisition relied on participants’ self-assessment after the training. While self-reported measures are subjective and may not accurately reflect actual learning, they remain valuable in capturing participants’ perceived confidence and shifts in attitude.^39 40^ This perceived confidence is particularly relevant, as it can influence participants’ willingness to apply these safety practices. In addition, this study did not assess the long-term retention or sustainability of the knowledge and skills gained from the training. Future implementations should prioritise objective assessments, such as pretraining and post-training tests, to better measure learning outcomes and examine the long-term retention or sustainability of the knowledge and skills gained from the training. Third, some results may be influenced by the stress generated by the simulation setting itself.^41^ However, stress-inducing factors such as time pressure, interruptions and workload are also common in real clinical environments and can similarly affect performance.^42 43^

To date, 70% of the permanent staff have completed the training, representing strong coverage, especially given the high turnover typically observed in healthcare. Temporary staff should also be systematically included, as they are associated with a higher incidence of medication errors compared with permanent staff.^34 35^ Although the full team coverage has not been achieved, this level of participation likely contributes to a positive snowball effect: because opioid preparation and administration require a double-check, there is a high probability that at least one person in each pair has already been trained, thereby reinforcing safe practices through peer influence.

A logical next step would be to implement this opioid RoH training on a rolling basis, ensuring continuous inclusion of all healthcare professionals based on staff turnover and patterns observed in MAE reports. Periodic refresher sessions would help maintain adherence to these safety principles, applied multiple times daily, and reduce the risk of drift over time. Based on the success of this training, the RoH has since been extended to other high-risk medications (antiparkinsonian drugs and fentanyl infusions RoHs). Beyond this, additional strategies should be explored to further mitigate medication-related risks. One such approach is bedside scanning, which allows real-time verification of the patient’s identity and the medication by matching wristband and drug barcodes.^44^ This technology can also flag documented allergies and expired medications, adding further safety layers.^35^ Despite its implementation challenges and initial costs, barcode scanning should be prioritised as it represents a gold standard in medication safety. Another complementary approach involves engaging patients as active participants in their own care, effectively serving as the final safety barrier before drug administration.^14^ However, this approach has limitations, particularly among older patients with cognitive impairment or in situations involving complex medications like opioids that require frequent adjustments. Finally, expanding the opioid RoH across multiple centres would help assess the reproducibility of the simulation in different clinical environments and strengthen the generalisability of its findings. Global perspectives highlight the importance of a balanced approach that safeguards appropriate access to opioids while mitigating risks through stewardship-oriented strategies focused on quality use, monitoring and education, thereby positioning the opioid RoH as a relevant tool to strengthen awareness and responsible practices among healthcare professionals.^45^,^47^

In conclusion, opioid administration errors are frequent and may have serious consequences, particularly for older, vulnerable patients. Fortunately, these errors are largely preventable and can be reduced through targeted, system-oriented interventions. The opioid RoH training appears to be a practical and scalable approach to reinforce the consistent application of the 5R rule and the double-check procedure, while enhancing participants’ ability to detect and prevent medication errors. Beyond individual learning, healthcare leaders are encouraged to integrate opioid-specific RoHs into continuing education programmes and to complement these trainings with system-level safeguards. By combining education with technological and organisational solutions, institutions can strengthen safety culture and move closer to the fundamental principle: first, do no harm.

## Supplementary material

10.1136/bmjoq-2025-003728 online supplemental file 1

## Data Availability

All data relevant to the study are included in the article or uploaded as supplementary information.
