# Supplementary material for: Opioid Room of Horrors: a simulation approach to strengthen drug administration safety
Source: BMJ Open Qual. 2025 Dec 25;14(4):e003728. doi: 10.1136/bmjoq-2025-003728 (PMC12742103; doi:10.1136/bmjoq-2025-003728)
Supplement: online supplemental file 1 [file bmjoq-14-4-s001.docx]

Appendix 1 – Steps of the simulation process

Briefing phase

The 10-minute introduction began with a welcome from the two educators, who invited participants to share their prior simulation experience and feelings about the exercise. Training objectives and simulation steps were presented, along with clear instructions encouraging participants to act authentically, without playing a role, and to follow the exercise to completion. Educators explained the room setup and scenario, and instructed participants to prepare and administer the opioid to the fictitious patient, assigning one participant to prepare the drug while the other performed the double-check. Participants were informed that they had 10 minutes to complete the task. They were encouraged to speak aloud to make their reasoning visible, to ask questions, and to continue the exercise even if an error was identified. They were informed that a 10-minute debriefing would follow the simulation. To preserve the integrity of the exercise, they were asked not to share details of the scenario and were assured that results would remain anonymous.

Simulation execution

During the simulation, the two educators closely observed the participants’ performance, focusing on their ability to identify errors, avoid hazards, and ensure the quality of the double-check process during opioid preparation. Each educator followed one participant: the first educator monitored the four errors and four hazards, while the second educator checked the double-check performance. An assessment form was completed in real time to document errors identified, hazards avoided, double-check performance and relevant observations. This form served as the basis for the structured debriefing phase.

Debriefing phase

The debriefing session began by acknowledging participants’ emotions to help relieve any stress from the experience. Both participants were invited to share their impressions, which were documented in the assessment form. Personalized feedback was provided to each pair based on the educators’ observations during the exercise. This included the identified errors and the hazards that were avoided, as well as an evaluation of the double-check performance. Particular attention was paid to participants’ moments of hesitation, doubt, or reactions that either contributed to or prevented errors. Educators reinforced the correct application of the 5R rule and the double-check process, emphasizing the importance of consistently applying these safety measures. They stressed that any deviation from these practices could lead to potentially harmful medication errors.
